# Field studies on low and fast compressibility of cement-mixed gravel in railway embankment construction

**DOI:** 10.1371/journal.pone.0288884

**Published:** 2023-08-18

**Authors:** Ungjin Kim, Dae Sang Kim

**Affiliations:** Advanced Railroad Civil Engineering Division, Korea Railroad Research Institute, Uiwang-si, Gyeonggi-do, South Korea; Texas A&M University System, QATAR

## Abstract

The cement-mixed gravel, which is used in the construction of railway embankment, is a relatively more expensive material compared to sandy soil. Having said that, it is used where small residual settlement is required for example, abutment transition zone, due to characteristic of higher strength than sandy soil. In this paper, the time-dependent settlement were evaluated using field data measured over a long period of time at two RSR (Reinforced Subgrade for Railways) construction sites using two different backfill materials (sandy soil and cement-mixed gravel). The embankment settlement with cement-mixed gravel as a backfill material was reduced by 78% compared to that with sandy soil. Further, the period for stabilizing the embankment before installation of the railway track was significantly reduced with cement-mixed gravel as a backfill material.

## Introduction

Recently, the use of concrete slab tracks has been extended from conventional ballast track to high-speed rail [1, 2]. The main difference between the two track types is the flexibility and ease in maintenance. In the case of gravel tracks, it is relatively frequent, but easy to repair the alignment error in the ballast layer by tamping and replenishing the ballast [3, 4]. Whereas, in the case of a concrete slab track, maintenance is relatively rare, but difficult and expensive because it is necessary to grout between the ballast and subgrade or adjust the rail height using fasteners. If a significant amount of maintenance work is required on a concrete slab track with an expensive initial construction cost, this is not in line with the purpose of constructing a concrete slab track to reduce LCC (Life Cycle Cost) by achieving maintenance free.

The main cause of the alignment change of railways on embankment subgrade is the long-term settlement of the ground and subgrade. The railway alignment changes not only with the accumulation of the passing weight of the train, but also time-dependent settlement due to embankment weight [5–8]. An embankment subgrade usually consists of granular soils such as sand or gravel. Time-dependent settlement of granular soil is caused by plastic strain on the potential surface [9, 10], crushing of particles [11, 12], and frictional sliding between intact grains [13]. Therefore, when constructing embankment subgrade for a railway, deformation characteristics such as time-dependent settlement according to the material characteristics are important. Various studies on settlement and strength characteristics according to the change in embankment material have been conducted through laboratory tests, such as behavior according to soil grain size [14–16] and cement content [17]. Some researches through field measurement have been conducted on bridge foundations [18] and soft ground improvement [19], but long-term field measurement studies on embankment materials are scarce. This is because of the increase in construction cost due to long-term measurement and the difficulty in protecting the sensor.

The cost effectiveness of cement-mixed gravel compared to that of sandy soil can be evaluated by decreasing the construction period and maintenance even though the material is expensive. Thus, in this study, the time-dependent settlement characteristics of sandy soil and cement mixed gravel applied to two construction sites for the railway embankment construction were compared and analyzed. Further, the stabilization periods of railway embankment before installation of railway track on the construction period at two construction sites of RSR (Reinforced Subgrade for Railways) were evaluated according to two different backfill materials.

## Stabilization period of RSR

**Fig 1:**
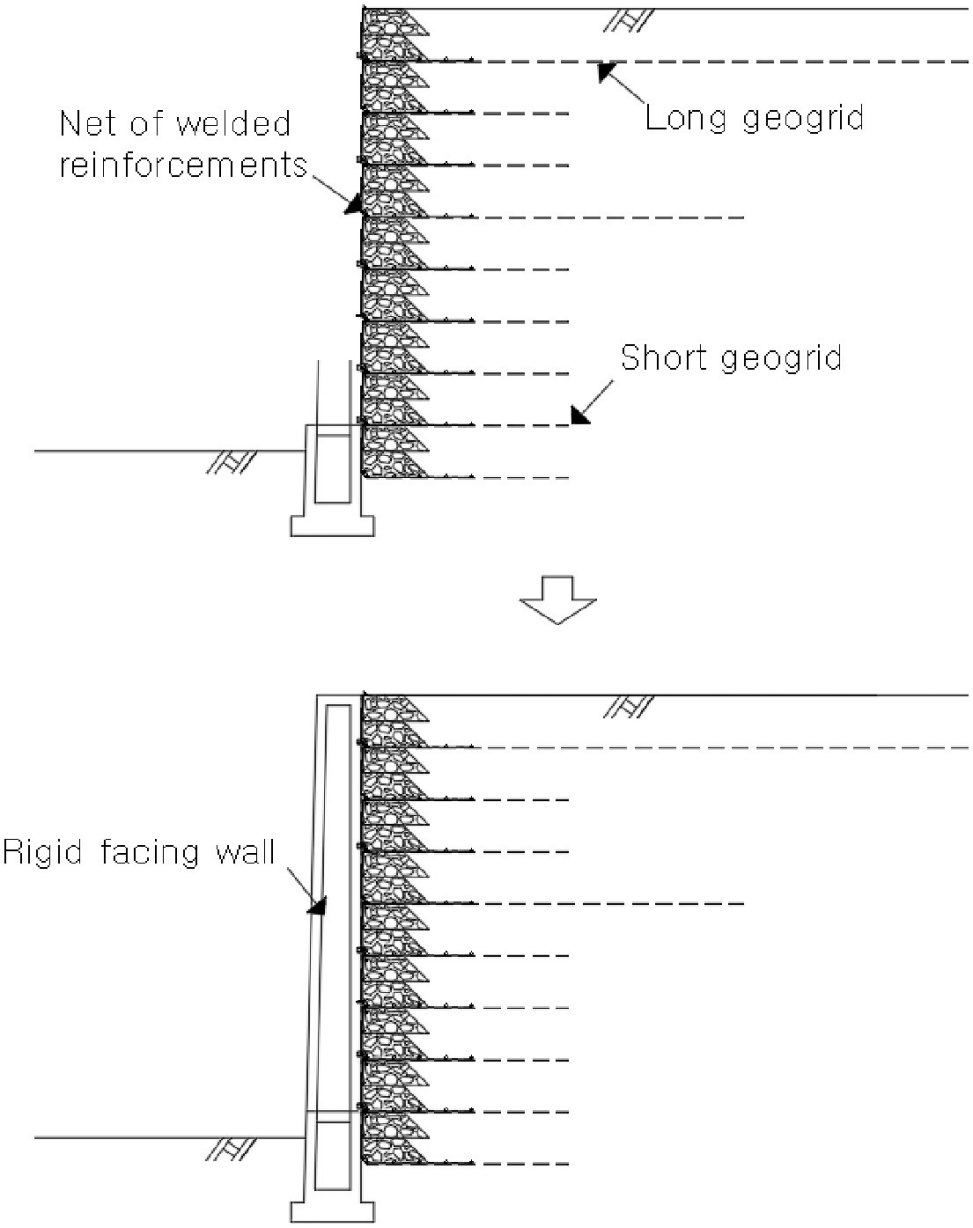
Embankment construction before the wall.

**Fig 2:**
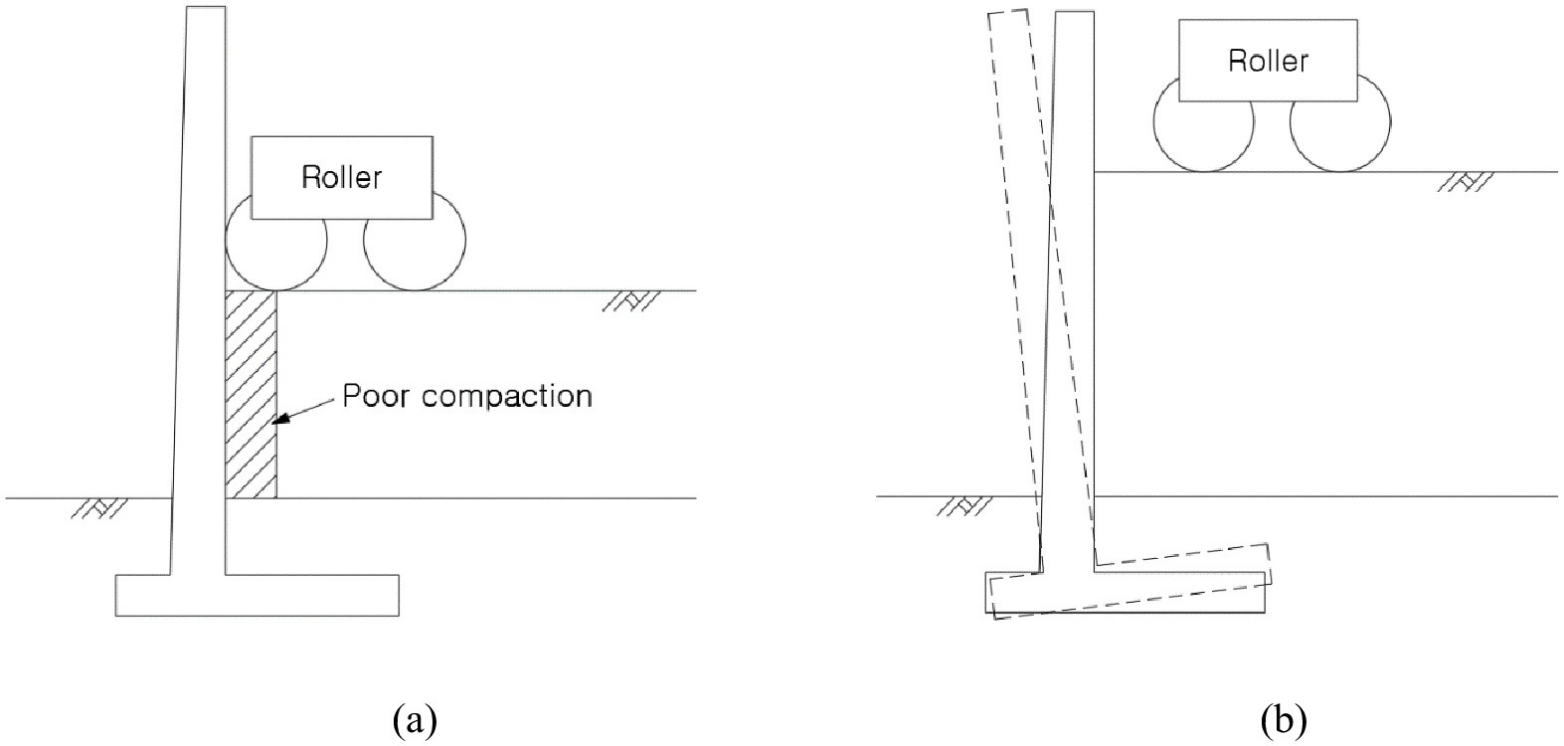
Poor compaction and wall overturning of traditional method. (a) Poor compaction, (b) Wall overturning.

**Fig 3:**
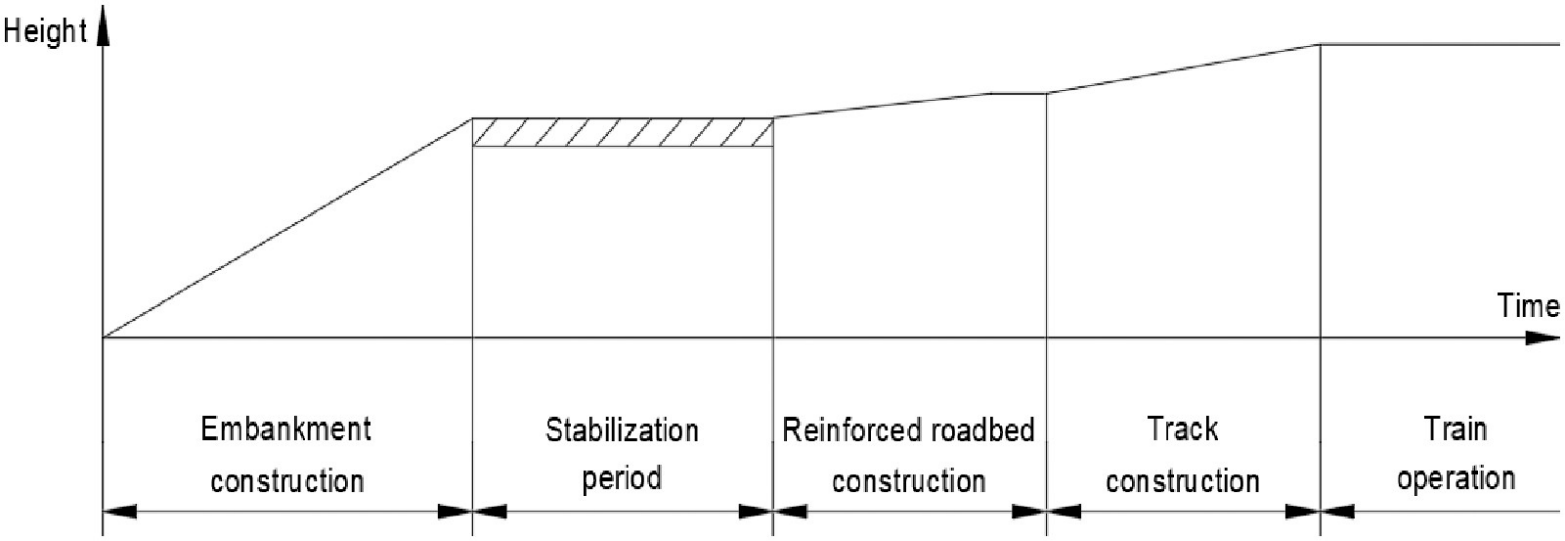
Concept of stabilization period.

The RSR is a construction method that minimizes the residual settlement through an embankment that is constructed before the rigid facing wall (Fig 1) [20–23]. The bearing capacity of the subgrade can be increased, and the residual settlement and horizontal earth pressure can be reduced through the integration of the reinforcement and the wall [24, 25]. In addition, this helps overcome the problems such as poor compaction in the embankment and wall overturning (Fig 2). To this end, the wall must be constructed only after the pre-constructed embankment has undergone a stabilization period that induces settlement by its weight (Fig 3). The stabilization period should be maintained until the settlement that satisfies the residual settlement stipulated in the design criteria, and it is strongly influenced by the materials that make up the ground and the embankment. Because no overburden load is added to the top of the embankment of the RSR during stabilization period, the time-dependent settlement caused by its own weight can be analyzed for each material from the settlement data during this period.

## Field measurement and analysis

### Case 1: Janghang line

#### Design and construction of RSR

**Fig 4:**
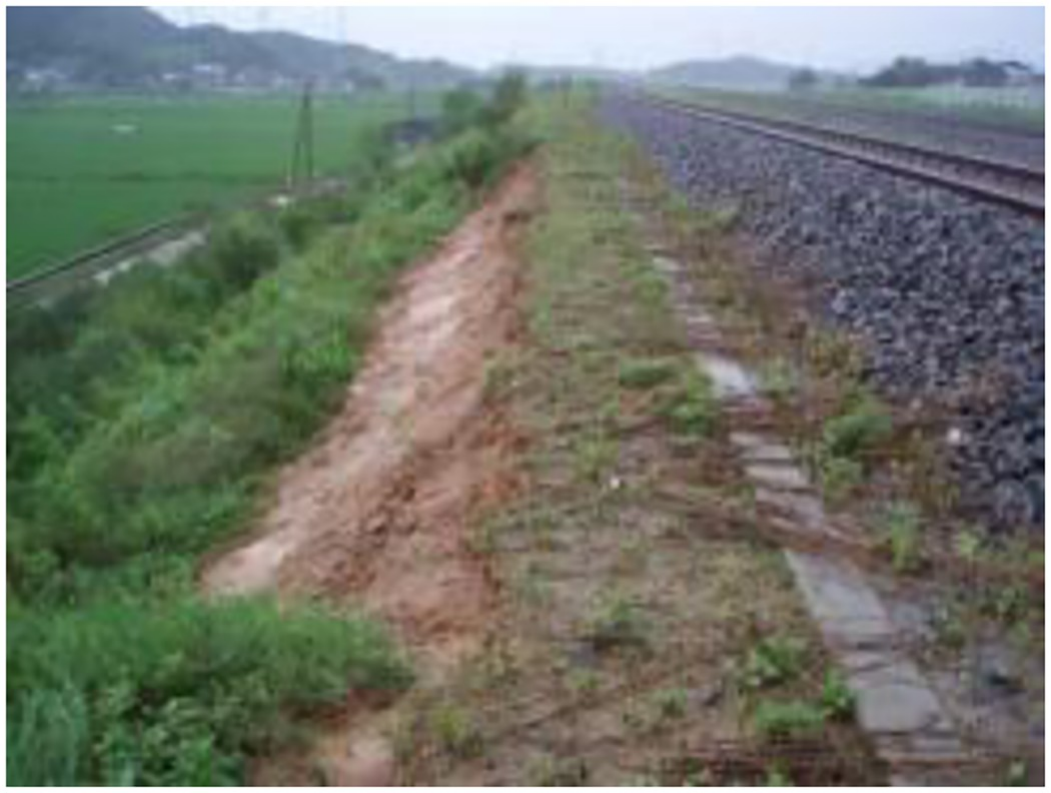
Slope failure of railway embankment by rainfall.

**Fig 5:**
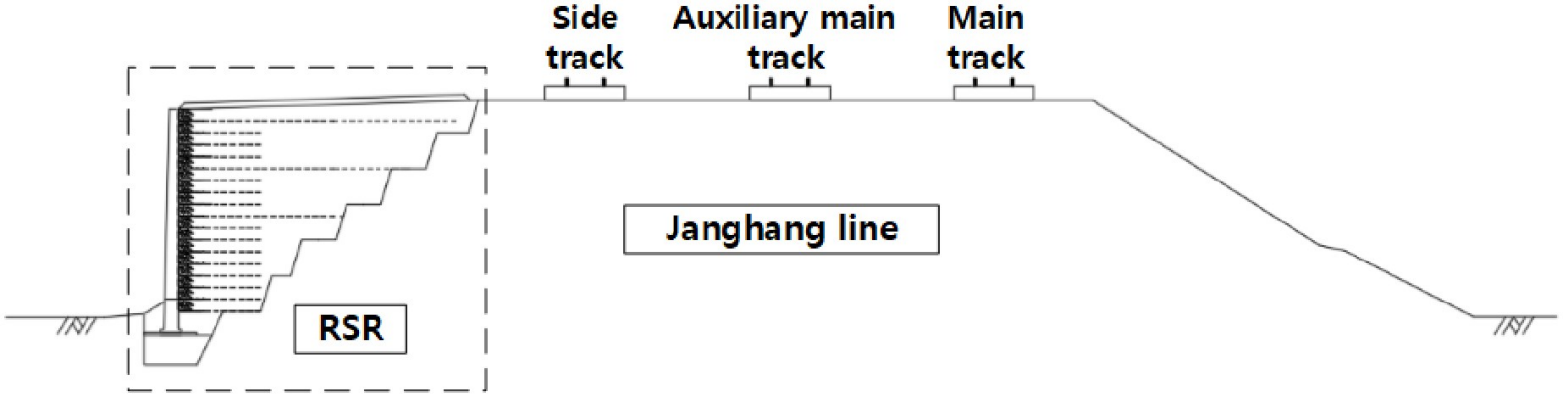
Section view of the Janghang railway line with RSR.

In the section of STA.80k760–80k800 of the Janghang Line, the subgrade slope was frequently damaged due to rain, and thus, it was temporarily restored using nonwoven fabric, as shown in Fig 4. The RSR, shown in Fig 5, was designed for the following two purposes: (1) complete slope recovery and (2) increase in the capacity of existing lines without additional sites.

Table 1 lists the results of the ground investigation for the design and construction of the RSR. The upper stratum up to 1.8 m from the surface was a landfill layer with silty sand with an N value of 3 to 7, and the stratum up to 3.5 m below was a sedimentary layer with silty clay with an N value of 5 to 8.

**Table 1:** Ground conditions.

Depth (m)	Thickness (m)	Layer description	SPT (Standard Penetration Test)
			N values (Number of blows/cm)
0.0 ~ 1.8	1.8	Land fill layer Soft to very soft silty sand	3/30 ~ 7/30
1.8 ~ 3.5	1.7	Sedimentary layer Soft to medium-stiff silty clay	5/30 ~ 8/30
3.5 ~ 5.7	2.2	Sedimentary layer Medium-stiff to stiff sandy gravel	31/30 ~ 21/30
5.7 ~ 23.5	17.8	Weathered soil layer Soft to very stiff silty sand	14/30 ~ 50/30

**Fig 6:**
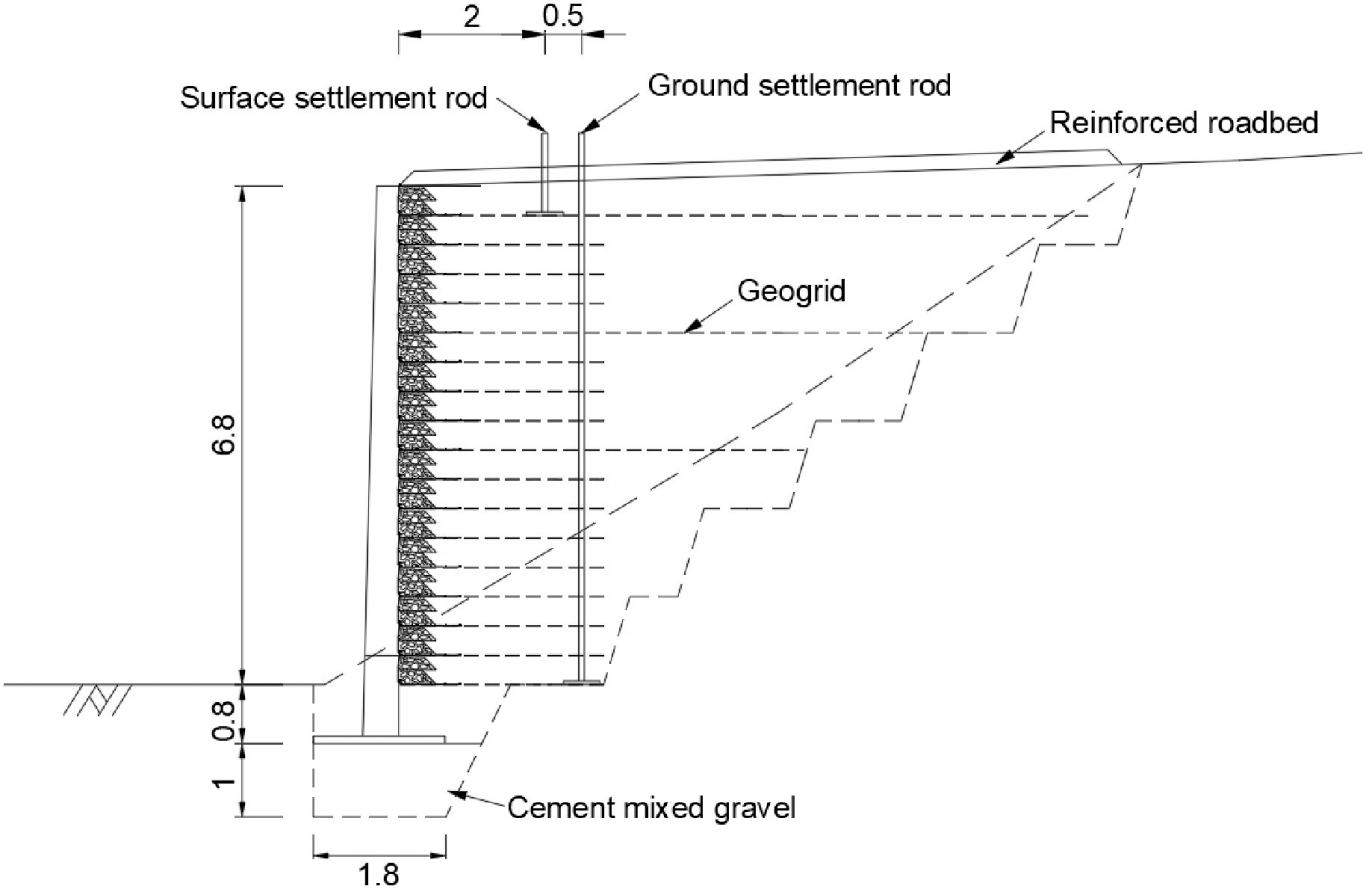
Section view of RSR design at Chang-hang railway line.

An RSR with a height of 7.6 m was designed on the slope of an existing operating line. Because the ground contained soft clay, the residual settlement was expected after the wall construction. Therefore, the 1.5 m-wide and 1.8-m deep ground was replaced with cement mixed gravel, as shown in Fig 6. The vertical spacing of the reinforcement installed in the embankment was 40 cm, and a short reinforcement of 2.8 m was applied, which is 40% of the height. The long reinforcements installed in the 9th, 13th, and 17th layers were applied to a sufficient length considering the safety, economic feasibility, and workability of the embankment.

**Fig 7:**
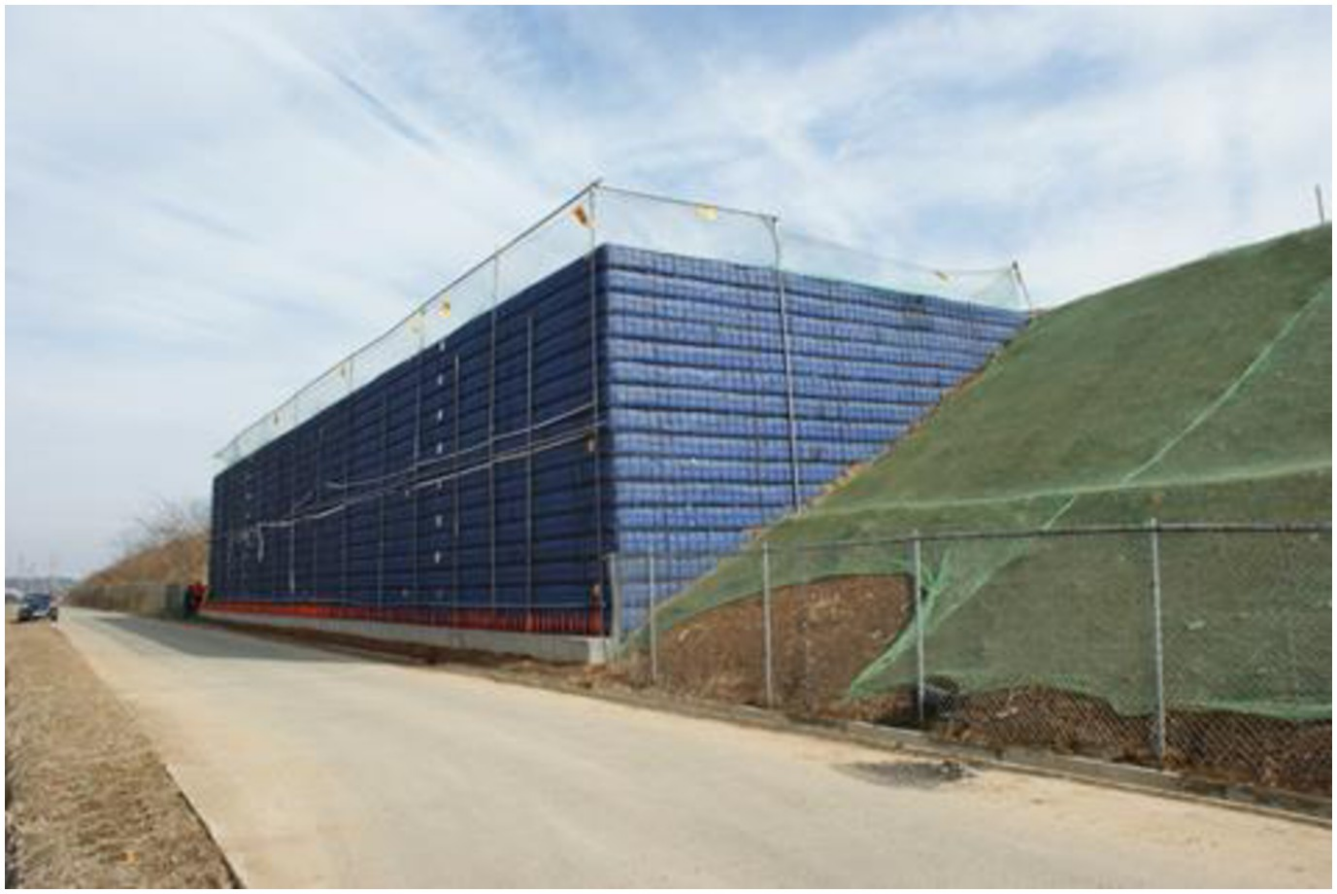
Embankment construction.

**Fig 8:**
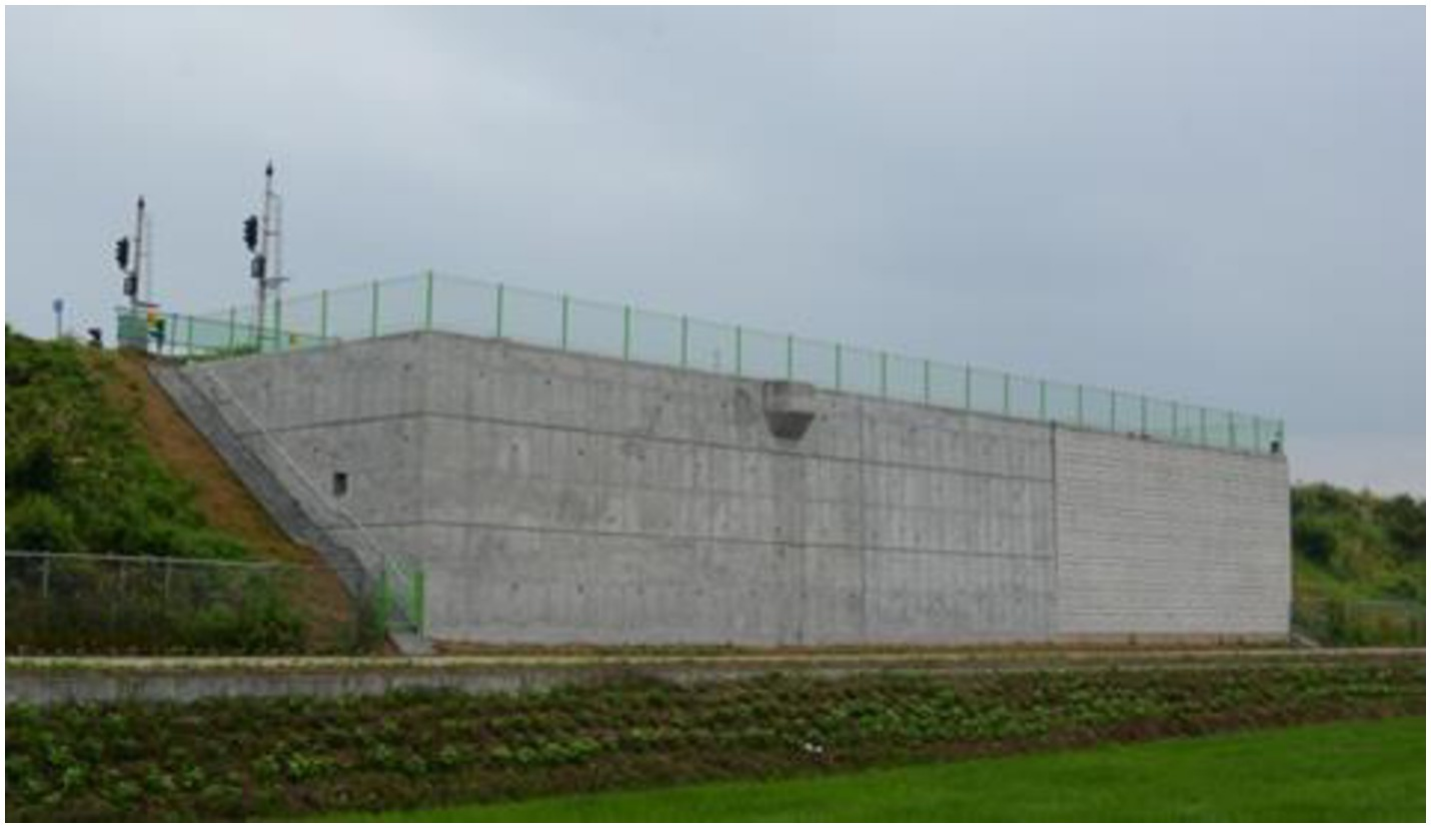
Rigid facing wall installation.

The SM(Silty sands) and SP(Poorly graded sands)-SM soil were used for the upper and lower embankments according to the unified soil classification system, respectively. The upper subgrade was constructed to satisfy the compaction criteria E_v2_(Second deformation modulus) > 80 MPa and E_v2_/E_v1_(First deformation modulus) < 2.3, and the lower subgrade was constructed to satisfy the compaction criteria E_v2_ > 60 MPa and E_v2_/E_v1_ < 2.7. Figs 7 and 8 show the RSR views after embankment and wall construction, respectively.

#### Long-term measurements and analyses of settlements

**Fig 9:**
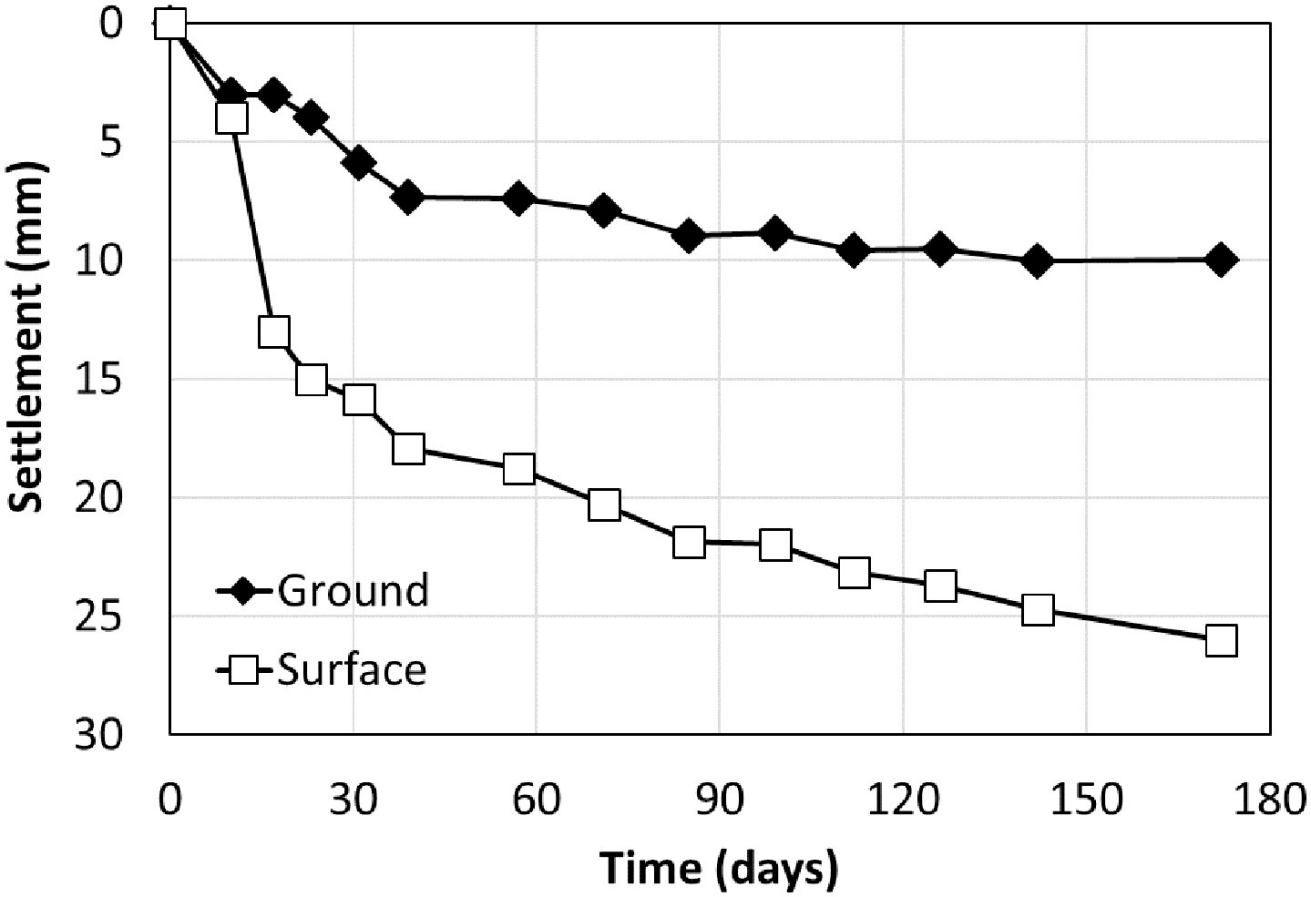
Settlement over time.

Once the embankment was completed, the settlements were measured for approximately six months on the ground and surface during the stabilization period before constructing the rigid facing wall. The ground and surface settlement rods were installed at a distance of 2.0 and 2.5 m from the outside of the RSR, respectively. Fig 9. shows the results of settlement measurements during a stabilization period of 172 days. The maximum settlement was 10.06 and 26.00 mm in the ground and the surface, respectively. Although sandy soil was used as the backfill material, the settlement increase in over time. This was similar to a previous study that showed creep deformation even in sandy soil [26–29].

Because the time-settlements curve showed hyperbolic form, the hyperbolic [30] and Hoshino [31] methods could be applied to predict the total settlement. Table 2 shows the calculations for the expected total and residual settlements of the ground and surface from the measurement results for 172 days. The settlement of the RSR embankment excluding the ground settlement, was 16–18 mm during the measurement period, and this value was negligible when the allowable residual settlement standard (including trainload) of the concrete slab track was 30 mm [32, 33]. The expected total settlement obtained with the Hoshino method was larger than that with the hyperbolic method.

**Table 2:** Measured, expected total, and residual settlements.

Settlements (mm)		Hyperbolic method	Hoshino method
Ground	Measured max.	10.06	10.06
	Expected total	12.66	13.02
	Residual	2.60	2.96
Surface	Measured max.	26.00	26.00
	Expected total	28.83	31.05
	Residual	2.83	5.05
Embankment		15.94	

**Fig 10:**
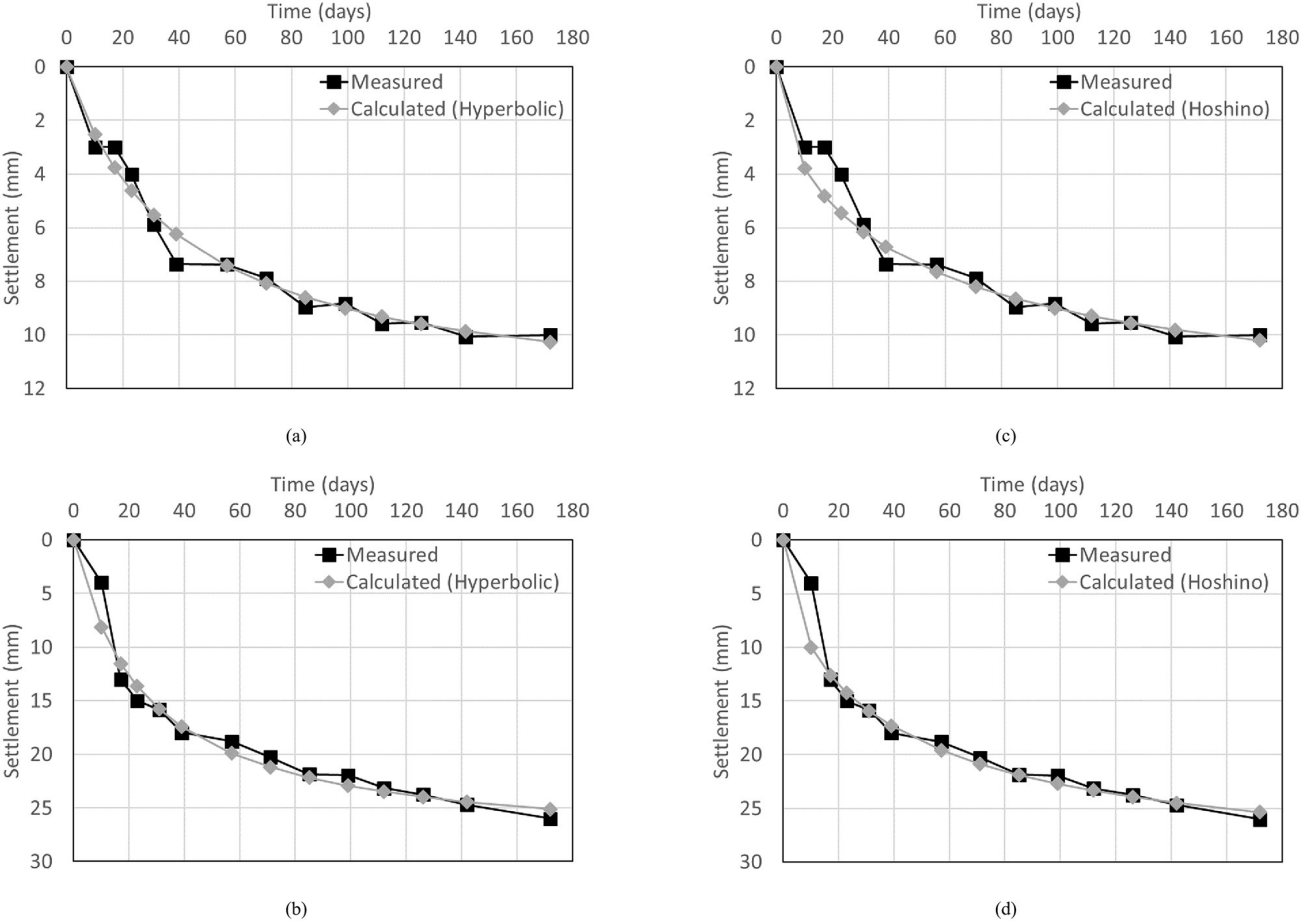
Comparison between the calculated and measured settlements. (a) Ground, Hyperbolic, (b) Surface, Hyperbolic, (c) Ground, Hoshino, (d) Surface, Hoshino.

The measured and calculated settlements over time were compared to evaluate the accuracy of each calculation method, as shown in Fig 10. The average error of the measured and calculated values for each measurement day was calculated, as summarized in Table 3. The average error (e_a_) is defined by Eq (1) as follows

$$e_{a}=\frac{\sum s_{c}-s_{m}}{n}$$ (1)

Where, s_c_ is the calculated settlement; s_m_ is the measured settlement; n is the number of measurements taken.

**Table 3:** Average errors depending on calculation method.

Average errors (mm)	Hyperbolic method	Hoshino method
Ground	0.38	0.53
Surface	0.97	0.87

The larger average errors of each calculation method were 0.53 mm for the ground in the Hoshino method and 0.97 mm for the surface in the hyperbolic method, showing a relatively large error in the surface settlement. The hyperbolic and Hoshino methods, which are used to predict the total and residual settlement of soft clay, can also be applied to the settlement of the RSR, and the error level was found to be under 1 mm.

### Case 2: Osong test line

#### Design and construction of RSR

**Fig 11:**
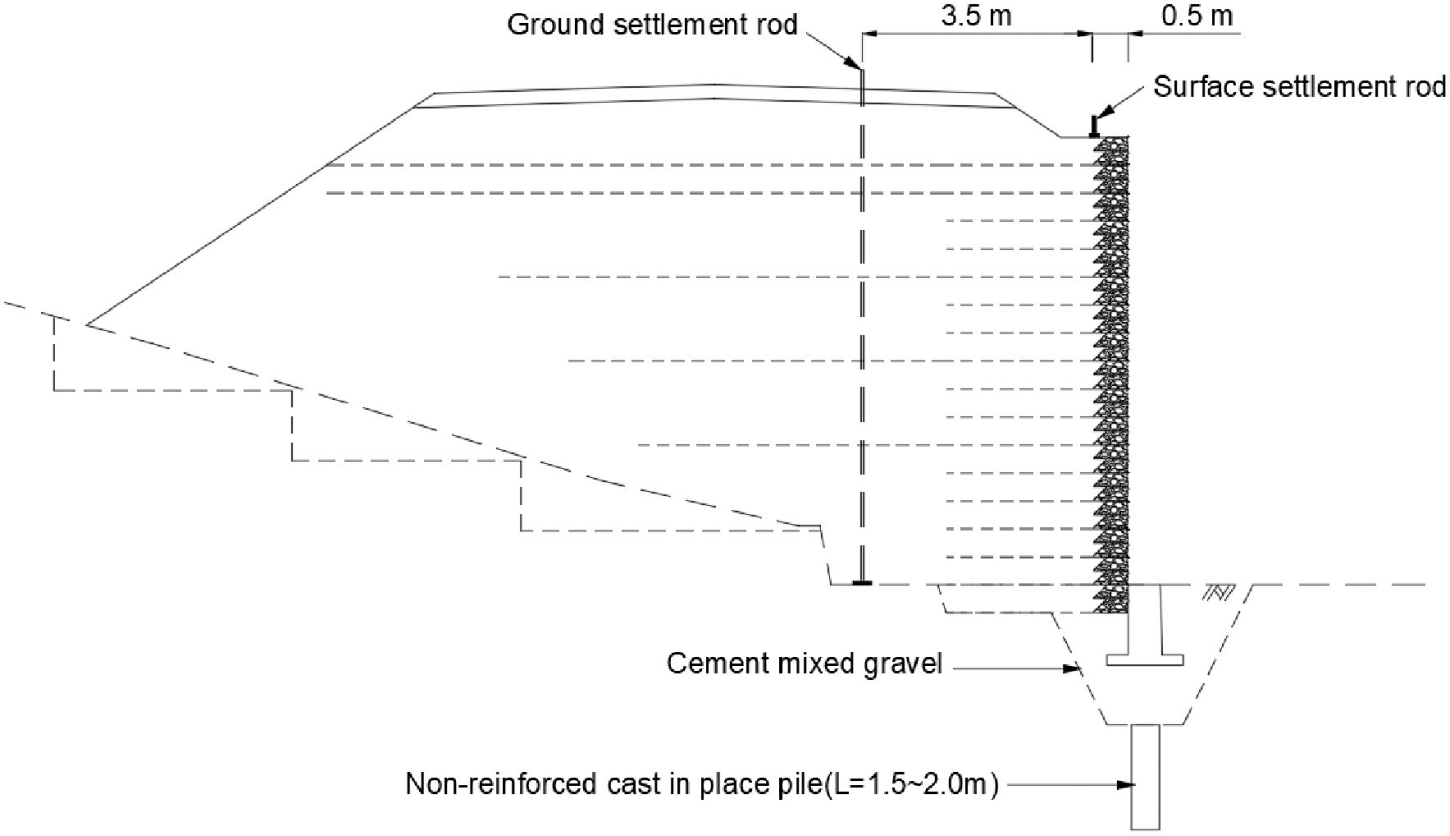
Section view of RSR design at Osong test line.

The abutment transition zone, previously designed as a reinforced concrete retaining wall, was changed to RSR according to the site conditions, as shown in Fig 11. Table 4 lists the results of the ground investigations. The stratum from 0 to 1.40 m was composed of clayey sand landfill layer, that from 1.40 to 3.70 m was composed of sandy clay sedimentary layer, and that below 3.70 m was composed of weathered soil and rock layer.

**Table 4:** Ground conditions.

Depth (m)	Thickness (m)	Layer descriptions	SPT N values (Number of blows/cm)
0 ~ 1.40	1.4	Landfill layer Medium stiff clayey sand	13/30
1.40 ~ 3.70	2.3	Sedimentary layer Stiff sandy clay	8/30 ~ 12/30
3.70 ~ 12.00	8.3	Weathered soil layer Soft to very stiff silty sand	16/30 ~ 50/9
12.00 ~ 15.00	3	Weathered Rock layer Very stiff weathered Granite	50/10 ~ 50/7

When designing the RSR, the short reinforcement length was 2.60 m (35% of the maximum wall height), and the length of the long reinforcement was installed to satisfy the criteria of circular failure, sliding, and overturning safety factors. The reinforcements were arranged at a vertical spacing of 40 cm, in accordance with the economic feasibility and workability. The upper layer of the ground (up to 2.0 m in depth) was replaced with cement-treated gravel to improve the soft layer, and a non-reinforced cast-in-place concrete pile with a length of 1.5 to 2.0 m was installed under the wall foundation.

**Fig 12:**
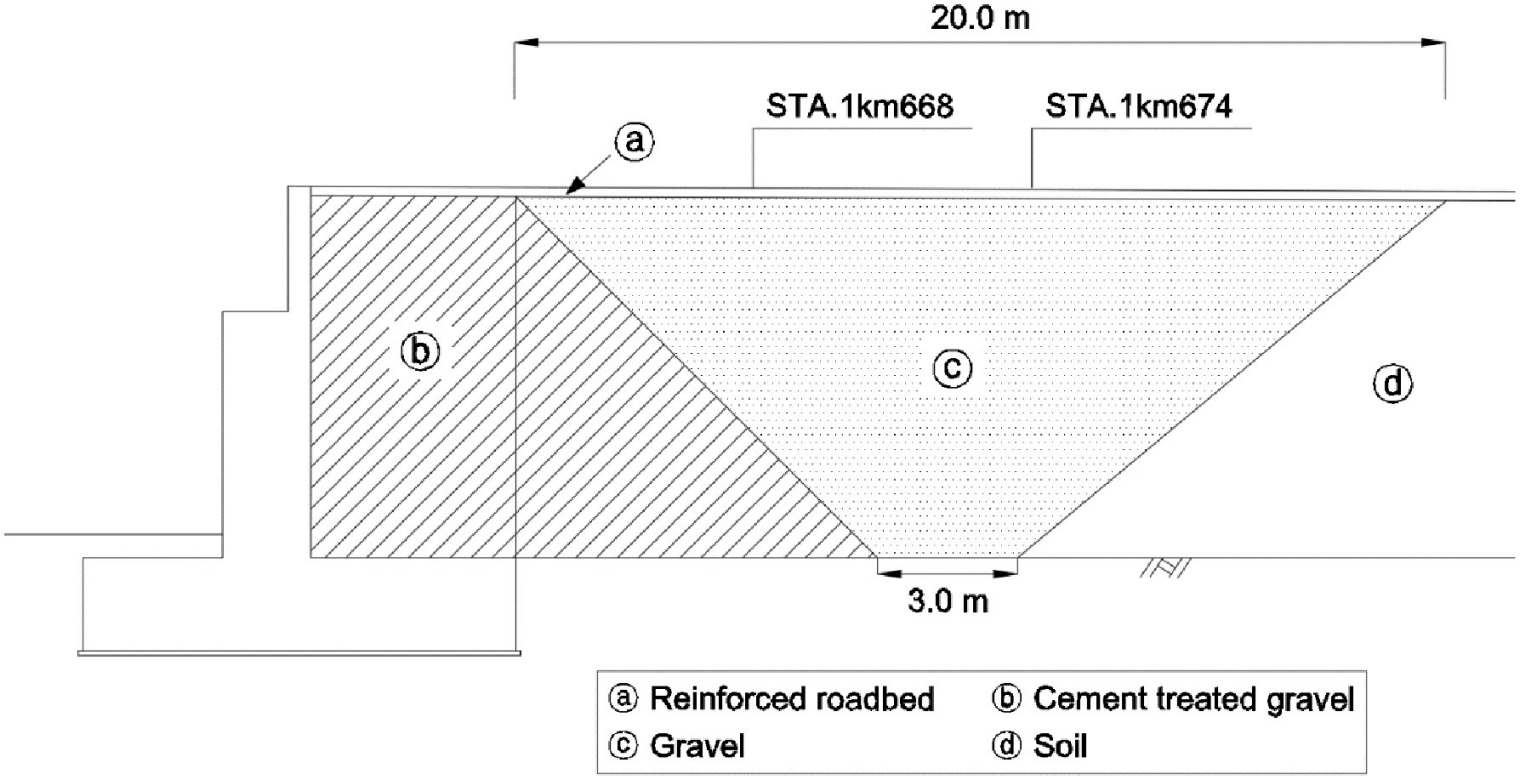
Backfill materials at transition zone.

An RSR with a maximum wall height of 7.5 m and a length of 156.0 m, including the abutment transition zone, was constructed. The abutment transition zone was constructed as shown in Fig 12, and different backfill materials were used for each section, according to the railroad design standard in Korea. Table 5 presents the compaction and quality standards for the materials used in each section. Cement mixed gravel with a weight ratio of 3% was applied to the transition zone ⓑ on the rear side of the abutment, and gravel with a maximum particle diameter of 63 mm was applied to the ⓒ zone.

**Table 5:** Properties and compaction conditions for backfill materials.

Zones	Materials	Properties and compaction conditions
ⓐ	Reinforced roadbed (Crushed gravel)	D_max_^a^ = 31.5 mm, E_v2_ ≧ 120 MPa, E_v2_/E_v1_ < 2.2
ⓑ	Cement treated gravel	D_max_ = 63 mm (3% cement) E_v2_ ≧ 120 MPa, E_v2_/E_v1_ < 2.2
ⓒ	Gravel	D_max_ = 63 mm, E_v2_ ≧ 80 MPa, E_v2_/E_v1_ < 2.2
ⓓ	Soil	Upper roadbed: E_v2_ ≧ 80 MPa, E_v2_/E_v1_ < 2.3 Lower roadbed: E_v2_ ≧ 60 MPa, E_v2_/E_v1_ < 2.7

^a^D_max_ = Maximum particle diameter.

#### Long-term measurements and analyses of settlements

**Fig 13:**
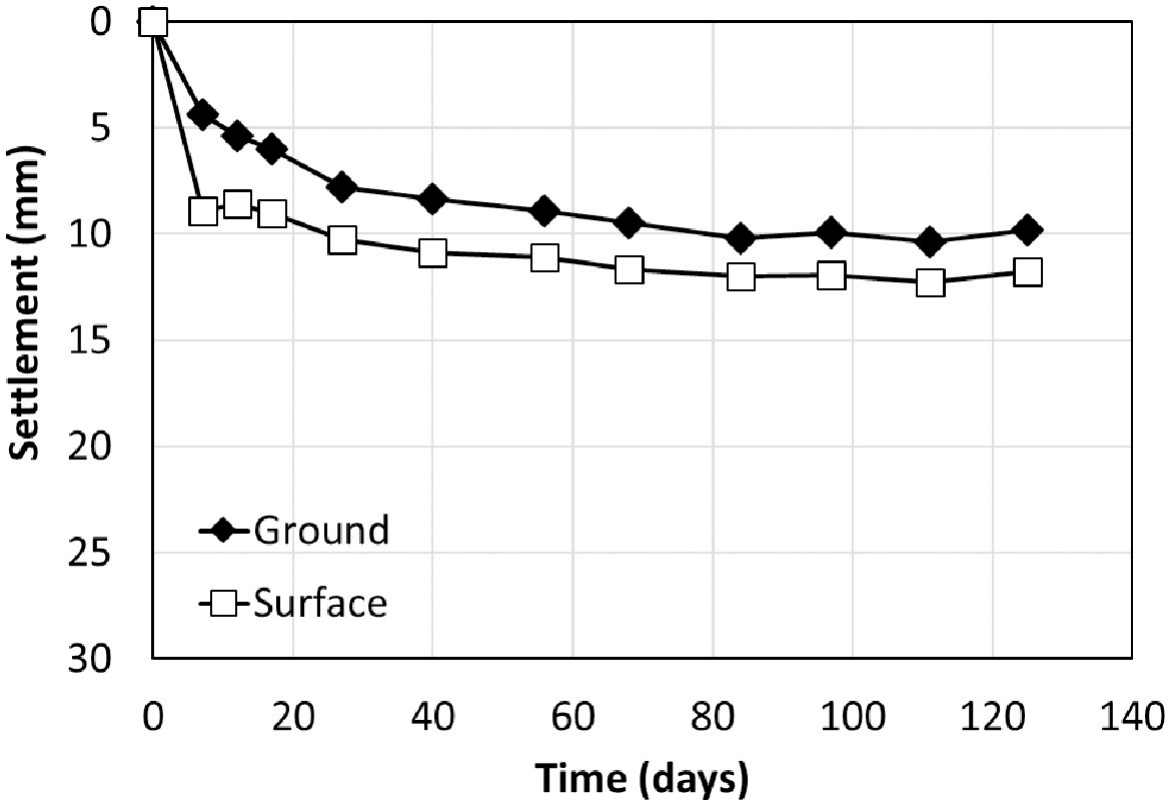
Settlement over time.

During the stabilization period of 125 days, settlement measurements were performed on the ground and surface. The ground and surface settlement rods were located at STA.1 km674 and STA.1 km668, respectively. The measurement location corresponds to the connection section, the ground settlement rod was installed at a distance of 4.0 m from the wall, and the surface settlement rod was installed at a distance of 0.5 m, as shown in Fig 11. The settlement measurements were performed from the completion of embankment construction until an overburden load was applied to the upper part of the subgrade for the subsequent process. During the stabilization period, the maximum settlement in the ground and surface was 10.38 and 12.26 mm, respectively, as shown in Fig 13.

Similar to Case 1, the total settlement was calculated using the hyperbolic and Hoshino methods. Table 6 shows the results of the final and residual settlements of the ground and surface from the measurements for 125 days. The settlement in the embankment, excluding the ground settlement, was extremely small (1.88 mm during the measurement period), and thus, it was considered to be negligible. Similar to Case 1, it was found that the final and residual settlements obtained with the Hoshino method were larger than those obtained with the hyperbolic method.

**Table 6:** Measured, expected total, and residual settlements.

Settlements (mm)		Hyperbolic method	Hoshino method
Ground	Measured max.	10.38	10.38
	Expected total	11.21	11.94
	Residual	0.83	1.56
Surface	Measured max.	12.26	12.26
	Expected total	12.53	12.64
	Residual	0.27	0.39
Embankment		1.88	

**Fig 14:**
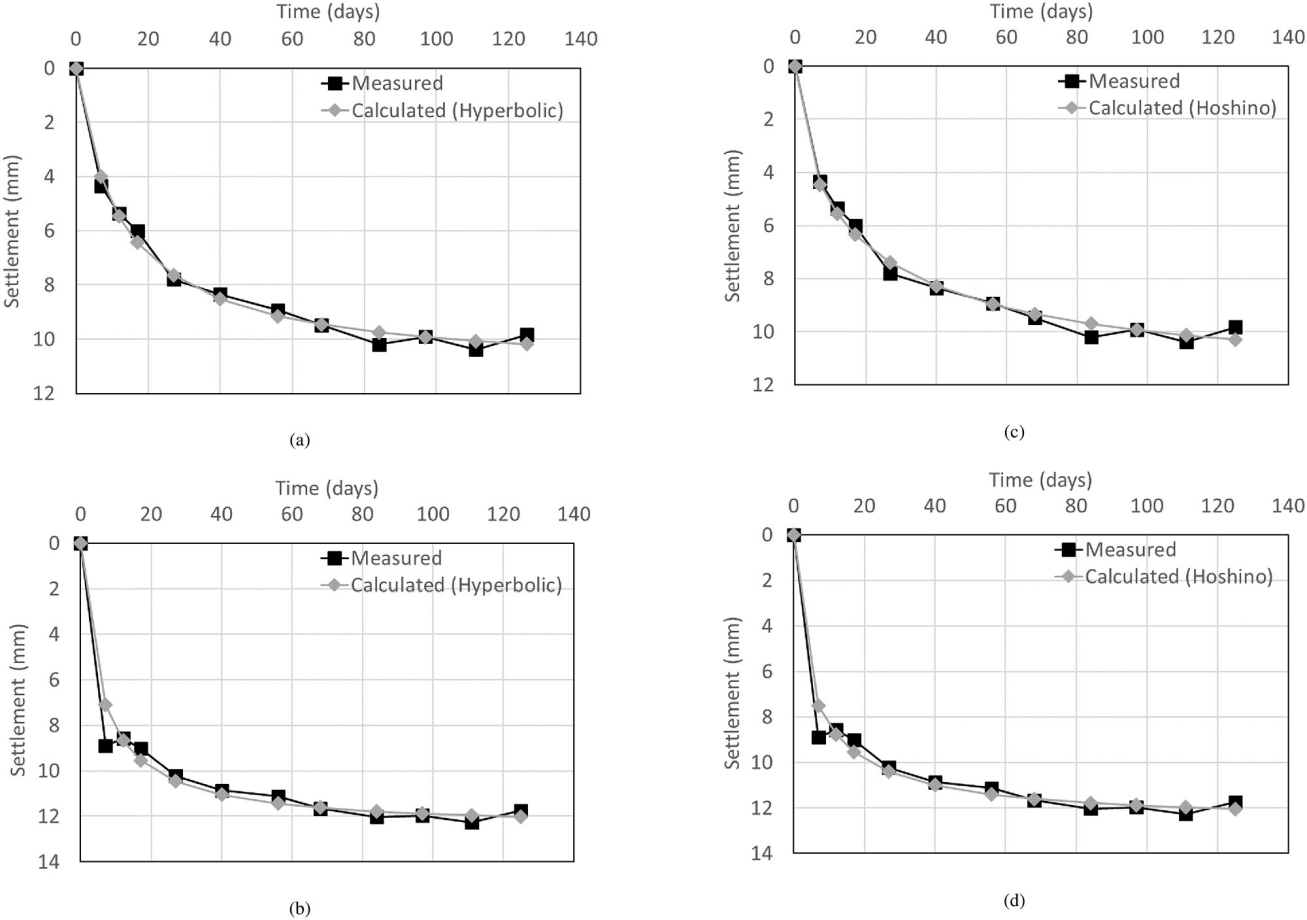
Comparison of calculated and measured settlements. (a) Ground, Hyperbolic, (b) Surface, Hyperbolic, (c) Ground, Hoshino, (d) Surface, Hoshino.

The measured and calculated settlements over time were compared to evaluate the accuracy of each calculation method, as shown in Fig 14. The average error of the measured and calculated values for each measurement day was calculated, as summarized in Table 7. The larger average errors of each calculation method were 0.23 mm for the ground in the Hoshino method and 0.37 mm for the surface in the hyperbolic method. The error was small (less than 0.5 mm) compared to that of the Case 1, wherein the backfill material was constructed with soil. In the case of RSR using gravel and cement-mixed gravel as backfill materials, the calculated settlement error in the roadbed was negligible.

**Table 7:** Average errors depending on calculation method.

Average errors (mm)	Hyperbolic method	Hoshino method
Ground	0.23	0.23
Surface	0.37	0.34

The hyperbolic and Hoshino methods were used to estimate the residual settlement of the RSR from the field measurement results of the two cases with different backfill materials (soil and gravel). Although the method has been applied to soft clay, the settlement of RSR was calculated with an accuracy within 1 mm of the average error because the settlement curves has the form of a hyperbola owing to the influence of time-dependent settlement of the backfill material and the ground containing soft clay.

## Evaluation of stabilization period

**Fig 15:**
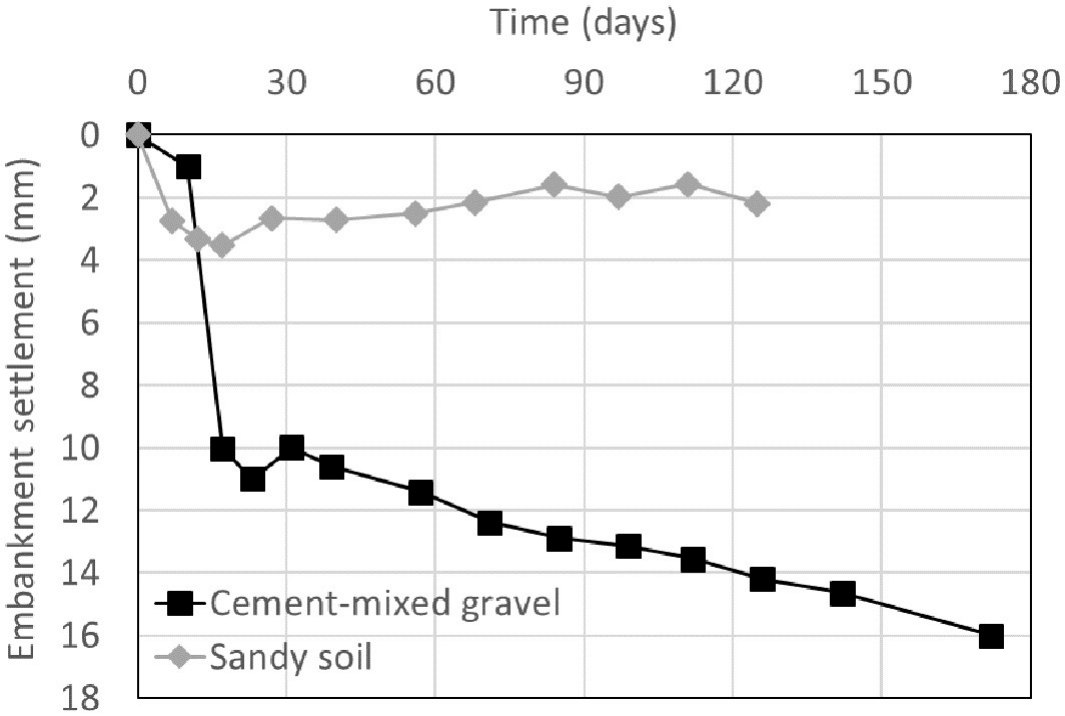
Embankment settlement over time.

To calculate the stabilization period, first, the allowable residual settlement should be determined. According to the Korean railroad design standards, the allowable residual settlement is applied differently for each track type. The allowable residual settlement of the gravel tracks was 100 mm, and that of the concrete tracks was 30 mm, including the trainload. The allowable residual settlement on the concrete track includes the expected settlement of 25 mm in the ground, embankment, and track after the construction of the roadbed and the settlement due to the train load (assuming 5 mm). Table 8 lists the calculation results of the stabilization period for the two cases based on the allowable residual settlement. In the Janghang line, the stabilization period was calculated as 10 to 17 days according to the settlement estimation method. Whereas in the Osong test line, immediate wall construction was possible. The main reason behind such a significant difference in the stabilization period between the two cases with similar RSR heights is the difference in the backfill material. In the two cases, the backfill material was applied in a different manner to SM soil and cement-mixed gravel in the Janghang and Osong test lines, respectively. The settlement in cement-mixed gravel is small owing to the increased strength and stiffness [34, 35] and the decreased creep [36, 37] occurring when the gravel is mixed with cement. Furthermore, as the aggregate diameter increases, the aggregates have less settlement and more self-compacting properties [38]. Therefore, the stabilization period decreased. Fig 15 shows the value obtained by subtracting the ground settlement from the surface settlement according to the embankment settlement over time. In the cement-mixed gravel, the embankment settlement increased up to 3.53 mm and then tended to converge to approximately 2 mm. However, in the sandy soil, the value constantly increased to a maximum of 16 mm. If the stabilization period cannot be sufficiently secured at a site with insufficient construction time, the stabilization period can be considerably reduced by using cement-mixed gravel as the backfill material. Furthermore, it can affect the construction period and the improvement of stability of the ballast layer.

**Table 8:** Stabilization period depending on calculation method.

Lines/Methods		Expected final settlement (mm)	Stabilization period (days)
Janghang line	Hyperbolic method	28.83	10
	Hoshino method	31.05	17
Osong test line	Hyperbolic method	12.53	0
	Hoshino method	12.64	0

## Conclusion

The cement-mixed gravel and sandy soil were applied to the RSR as a backfill material, and the ground and surface settlements were measured at the two railway construction sites for 172 and 125 days, respectively. The conclusions of the study are summarized below.

1. The time-dependent settlement of embankment with cement-mixed gravel as a backfill material was reduced by 78% compared to that with sandy soil. While the time-settlement curve converges to a certain level in cement-mixed gravel case, it constantly increases in the sandy soil case during the stabilization period.
2. The stabilization period evaluated from surface settlement of railway embankment with cement-mixed gravel as a backfill material was significantly decreased. Thus, the RSR with cement-mixed gravel as a backfill material could be effectively applied to railway sites where rapid construction is needed such as restoration of collapsed subgrade.
3. The embankment settlement defined by “the difference between surface and ground settlement” was converged within 17days in the railway embankment with cement-mixed gravel as a backfill material. However, in the railway embankment settlement with sandy soil, the settlement-time curve reached the infection point at 23 days after embankment construction since then the settlement gradually increased. It is considered that the cement-mixed gravel as a backfill material has a good performance in the aspect of settlement.

## References

[1] Hong C.K., Yang S.C., Kim Y.T., A Study on the Criteria of Settlement in Concrete Slab track, Journal of the Korean Society for Railway. 2007;10(3):355–364.

[2] Steenbergen M., Metrikine A.V., Esveld C. Assessment of design parameters of a slab track railway system from a dynamic viewpoint, Journal of Sound and Vibration. 2007;306(1–2):361–371. doi: 10.1016/j.jsv.2007.05.034

[3] Furukawa A., Recent tendencies in ballasted track maintenance, Quarterly Report of RTRI. 2016;57(2):80–84. doi: 10.2219/rtriqr.57.2_80

[4] Huang J.J., Su Q., Zhao W.H., Li T., Zhang X.X., Experimental study on use of lightweight foam concrete as subgrade bed filler of ballastless track, Construction and Building Materials. 2017;149:911–920. doi: 10.1016/j.conbuildmat.2017.04.122

[5] Auersch L., Dynamics of the railway track and the underlying soil: the boundary-element solution, theoretical results and their experimental verification, Vehicle System Dynamics. 2005;43(9):671–695. doi: 10.1080/00423110412331307663

[6] Bian X., Jiang H., Chang C., Hu J., Chen Y., Track and ground vibrations generated by high-speed train running on ballastless railway with excitation of vertical track irregularities, Soil Dynamics and Earthquake Engineering. 2015;76:29–43. doi: 10.1016/j.soildyn.2015.02.009

[7] Chen R.P., Jiang P., Ye X.W., Bian X.C., Probabilistic analytical model for settlement risk assessment of high-speed railway subgrade, Journal of Performance of Constructed Facilities. 2016;30(3):04015047. doi: 10.1061/(ASCE)CF.1943-5509.0000789

[8] Guo Y., Wanming Z., Long-term prediction of track geometry degradation in high-speed vehicle–ballastless track system due to differential subgrade settlement, Soil Dynamics and Earthquake Engineering. 2018;113:1–11. doi: 10.1016/j.soildyn.2018.05.024

[9] Lade P.V., Creep effects on static and cyclic instability of granular soils, Journal of Geotechnical Engineering. 1994;120(2):404–419. doi: 10.1061/(ASCE)0733-9410(1994)120:2(404)

[10] Lade P.V., Liu C.T., Experimental study of drained creep behavior of sand, Journal of engineering mechanics. 1998;124(8):912–920. doi: 10.1061/(ASCE)0733-9399(1998)124:8(912)

[11] Hardin B.O., Crushing of soil particles, Journal of geotechnical engineering. 1985;111(10):1177–1192. doi: 10.1061/(ASCE)0733-9410(1985)111:10(1177)

[12] Lade P.V., Karimpour H., Static fatigue controls particle crushing and time effects in granular materials, Soils and foundations. 2010;50(5):573–583. doi: 10.3208/sandf.50.573

[13] Karimpour H., Lade P.V., Time effects relate to crushing in sand, Journal of Geotechnical and Geoenvironmental Engineering. 2010;136(9):1209–1219. doi: 10.1061/(ASCE)GT.1943-5606.0000335

[14] Qi S., Cui Y.J., Chen R.P., Wang H.L., Lamas-Lopez F., Aimedieu P., et al. Influence of grain size distribution of inclusions on the mechanical behaviours of track-bed materials, Géotechnique. 2020;70(3):238–247. doi: 10.1680/jgeot.18.P.047

[15] Wani K.M.N., Mir B.A., Unconfined compressive strength testing of bio-cemented weak soils: a comparative upscale laboratory testing, Arabian Journal for Science and Engineering. 2020;45(10):8145–8157. doi: 10.1007/s13369-020-04647-8

[16] Wen R., Tan C., Wu Y., Wang C., Grain size effect on the mechanical behavior of cohesionless coarse-grained soils with the discrete element method, Advances in Civil Engineering. 2018. doi: 10.1155/2018/4608930

[17] Kenai S., Bahar R., Benazzoug M., Experimental analysis of the effect of some compaction methods on mechanical properties and durability of cement stabilized soil, Journal of Materials Science. 2006;41(21):6956–6964. doi: 10.1007/s10853-006-0226-1

[18] Yang Q., Leng W.M., Zhang S., Nie R.S., Wei L.M., Zhao C.Y., et al. Long-term settlement prediction of high-speed railway bridge pile foundation, Journal of Central South University. 2014;21(6):2415–2424. doi: 10.1007/s11771-014-2195-x

[19] Zhou S., Wang B., Shan Y., Review of research on high-speed railway subgrade settlement in soft soil area, Railway Engineering Science. 2020;28:129–145. doi: 10.1007/s40534-020-00214-x

[20] M. Tateyama, O. Murata, F. Tatsuoka, Earth retaining wall with short geotextile and a rigid facing, Proceedings of the 12th ICSMFE. Vol. 2. 1989:1311–1314.

[21] F. Tatsuoka, Roles of facing rigidity in soil reinforcing, Keynote Lecture, Proc. Int. Symp. on Earth Reinforcing Practice (IS Kyushu’91). 1992: 831–870.

[22] Tatsuoka F., Tateyama M., Mohri Y., Matsushima K., Remedial treatment of soil structures using geosynthetic-reinforcing technology, Geotextiles and Geomembranes. 2007;25(4–5):204–220. doi: 10.1016/j.geotexmem.2007.02.002

[23] Tatsuoka F., Tateyama M., Uchimura T., Koseki J, Geosynthetic-reinforced soil retaining walls as important permanent structures 1996–1997 mercer lecture, Geosynthetics International. 1997;4(2):81–136. doi: 10.1680/gein.4.0090

[24] Kim U.J., Kim D.S., Load sharing characteristics of rigid facing walls with geogrid reinforced railway subgrade during and after construction, Geotextiles and Geomembranes. 2020;48(6):940–949. doi: 10.1016/j.geotexmem.2020.08.002

[25] Kim U., Kim D.S., Evaluation of Deformation Characteristic of Railway Subgrade Using Reinforced Rigid Walls with Short Reinforcement under Repetitive and Static Loads, Applied Sciences. 2021;11(8):3615. doi: 10.3390/app11083615

[26] Augustesen A., Liingaard M., Lade P.V., Evaluation of time-dependent behavior of soils, International Journal of Geomechanics. 2004;4(3):137–156. doi: 10.1061/(ASCE)1532-3641(2004)4:3(137)

[27] Karimpour H, Lade P.V., Creep behavior in Virginia Beach sand, Canadian Geotechnical Journal. 2013;50(11):1159–1178. doi: 10.1139/cgj-2012-0467

[28] J. Su, S. Wang, Experimental Study on Creep Characteristics of Saturated Sandy Soil with Different Fines Content, IOP Conference Series: Earth and Environmental Science. 2021;719(4):042070.

[29] Tatsuoka F., Ishihara M., Di B.H., Kuwano R., Time-dependent shear deformation characteristics of geomaterials and their simulation, Soils and foundations. 2002;42(2):103–129. doi: 10.3208/sandf.42.2_103

[30] Tan T.S., Inoue T., Lee S.L., Hyperbolic method for consolidation analysis, Journal of geotechnical engineering. 1991;117.11:1723–1737. doi: 10.1061/(ASCE)0733-9410(1991)117:11(1723)

[31] Hoshino K., Problems of foundations in recent years, Society of civil engineering (Japanese). 1962;47.7:63–67.

[32] Korea Railway Network Authority, Railway design standard for roadbed, Ministry of Land, Infrastructure and Transport (in Korea). 2016.

[33] C. Göbel, K. Lieberenz, Handbuch Erdbauwerke der Bahnen, Eurailpress Tetzlaff-Hestra. 2004.

[34] Jiang N., Wang C., Wang Z., Li B., Liu Y.A., Strength characteristics and microstructure of cement stabilized soft soil admixed with silica fume, Materials. 2021;14(8):1929. doi: 10.3390/ma14081929 33921456 PMC8069276

[35] Kongsukprasert L., Tatsuoka F., Takahashi H., Effects of curing period and stress conditions on the strength and deformation characteristics of cement-mixed soil, Soils and foundations. 2007;47(3):577–596. doi: 10.3208/sandf.47.577

[36] Delfosse-Ribay E., Djeran-Maigre I., Cabrillac R., Gouvenot D., Factors affecting the creep behavior of grouted sand, Journal of geotechnical and geoenvironmental engineering. 2006;132(4):488–500. doi: 10.1061/(ASCE)1090-0241(2006)132:4(488)

[37] Venda Oliveira P.J., Correia A.A., Garcia M.R., Effect of organic matter content and curing conditions on the creep behavior of an artificially stabilized soil, Journal of materials in civil engineering. 2012;24(7):868–875. doi: 10.1061/(ASCE)MT.1943-5533.0000454

[38] Mohaddes Pour M., Razavi Taheri S.S., d. Shock and Vibration, 2021;2021:7612956. doi: 10.1155/2021/7612956

